# Supplementary material for: Self-strengthening biphasic nanoparticle assemblies with intrinsic catch bonds
Source: Nat Commun. 2021 Jan 4;12:85. doi: 10.1038/s41467-020-20344-4 (PMC7782701; doi:10.1038/s41467-020-20344-4)
Supplement: Supplementary file 1 — Supplementary Information [file 41467_2020_20344_MOESM1_ESM.pdf]

Supplementary Information for

Self-strengthening biphasic nanoparticle assemblies with intrinsic catch bonds

Kerim C. Dansuk[1], Sinan Keten[1,2]
[1] Department of Mechanical Engineering, Northwestern University, 2145 Sheridan Road, Evanston, IL 60208, USA

[2] Department of Civil & Environmental Engineering, Northwestern University, 2145 Sheridan Road, Evanston, IL 60208, USA

The PDF file includes:

- Supplementary Text
- **Supplementary Fig. 1.** X-shaped nanoparticle geometry and its dimensions
- **Supplementary Fig. 2.** Energy landscape of the SBS interaction.
- **Supplementary Fig. 3**. Possible DNA origami design of the X-shaped nanoparticle.
- **Supplementary Table 1**. Nanoparticle Dimensions

Other Supplementary Material for this manuscript includes the following:

- **Supplementary Movie 1.** Trajectory of a sample MD simulation run of the dimer pulled with 140 pN, dimer separates with low affinity unbinding.
- **Supplementary Movie 2.** Trajectory of a sample MD simulation run of the dimer pulled with 180 pN, dimer separates with high affinity unbinding.
- **Supplementary Movie 3.** Replica exchange MD Simulation of self-assembly of nanoparticles

Supplementary Text

The X-shaped nanoparticle consists of 10 members that are connected with hinge joints. In Supplementary Figure 1, member types a,b and c indicate the structure members and type d indicate the switch members. The bonded interactions between the beads are modelled as

harmonic springs, with $5000\ kcal/(mol\text{Å}^2)$ stiffness. To ensure that the members are straight, a harmonic angle potential with a spring coefficient of $1000\ kcal/(mol\ radian^2)$ is used. Each bead has a mass of 100 g/mol.

In the main text, we described that in the open conformation, the SBS are closer to the center of the particle compared to the PBS. In closed conformation, both PBS and SBS are on the same horizontal line. Here, we will describe how we determine the dimensions of the members to satisfy this relation. In the open conformation, the switch is angled and in equilibrium, thus the distance h between the SBS and the center of the particle equals to $x_{o,S}$, the equilibrium distance of the switch interaction. The intramolecular angle in the open conformation, θ_{LA} should satisfy the following relation

$$\cos(\theta_{LA}) = \frac{a^2+x_{o,S}^2-b^2}{2ah}. \qquad [S1]$$

When $\theta = \theta_{LA}$, The difference in vertical distance of PBS and SBS with respect to the center of the particle is given by

$$\Delta x = (a+b)\ \cos\theta_{LA} - x_{o,S}. \qquad [S2]$$

When two nanoparticles interact in their open state, complementary PBS of the dimer are at the equilibrium distance of the PBS interaction $x_{o,PBS}$ and the SBS are at a distance of $x_{o,PBS} + 2\Delta x$. As shown in Supplementary Fig. 2, the corresponding energy value of E_{SBS} is very small at this distance, which makes the assumption that SBS interaction is negligible in the LA state.

During the open to closed conformation transition, the interaction of the switch breaks, and the switch members become straight. At this point, h equals to $2d$, i.e. the length of the two switch

members. The intramolecular angle at the closed conformation, θ_{HA} should satisfy the following relation

$$\cos(\theta_{HA}) = \frac{a^2+(2d)^2-b^2}{2ah}. \qquad [S3]$$

Hence, for PBS and SBS to be on the same horizontal plane, the following relation must be true at θ_{HA}

$$h = (a+b)\ \cos\theta_{HA} = 2d. \qquad [S4]$$

The dimensions used in this work are listed in Supplementary Table 1. In conclusion, by varying the dimensions, we can control the changes in the intermolecular interactions between the conformational states.

X-shaped nanoparticle using DNA origami

Our nanoparticle design offers simple thought experiments that generate important insights for building nanoparticles with catch bonding interfaces, however, many more steps are needed to go from this design to a synthetic system. In regard to this, we offer some rationale as to how one might go about creating various components of the nanoparticle, taking the versatile framework of DNA origami as an example. As shown in Supplementary Fig. 3, we highlight four main components of the nanoparticle: stiff members, hinges, switch and the interaction sites.

Stiff members can be created from a bundle of interconnected double-stranded DNA helices organized in a honeycomb configuration (Supplementary Fig. 3B). Helix-bundles reportedly reach persistence lengths that exceed 2000 nm, thus will behave as stiff rods in the relevant scales to our system[1]. These members can be connected from both ends by several flexible single-stranded DNA

(ssDNA) scaffold connections arranged in a line to form the hinge rotation axis (Supplementary Fig. 3C). ssDNA exhibits a persistence length of ~2 nm, thus it is expected the system would be compliant at the hinge joints. Moreover, it has been shown in various DNA origami structures that ssDNA hinge can rotate flexibly over a range of angles[2].

In our X-shape nanoparticle design, the two-membered angled switch served two purposes. First, it provided an energy barrier between two conformations. Second, it limited further deformation when system transitioned to the closed state. These effects can also be created with a DNA loop structure, i.e. a hairpin. Hairpins are ssDNA or RNA sequences with complementary base pairs. In absence of force, these base pairs bind with hydrogen bonds form a loop (Supplementary Fig. 3D). When the hairpin is under force, hydrogen bonds break and cause the chain to unfold and increase its extensibility. Thus, hairpin creates an energy barrier for conformational change due to its hydrogen bonds and prevents elongation after the chain elongates to its contour length.

Lastly, DNA overhangs can be used to attain different rate kinetics in primary and secondary binding sites at the tips of the X-shaped nanoparticle. Overhangs in this case are ssDNA that have sticky ends. As shown in Supplementary Fig. 3E, complementary sticky ends (shown in red) form hydrogen bonds and form the bridge between two nanoparticles. On the other hand, inner bases (shown in grey) play the role of flexible linkers. For a system to show catch bond behavior, we showed that energy landscapes of primary, secondary and switch energy landscapes differ in well depth and width. Overhangs enable us to tune energy landscapes since sticky end length and base sequence effects the strength of DNA sticky end links[3]. Thus, increasing the complementary bases would deepen the energy well, and the flexible linkers could offer compliance to tune the curvature of the energy landscape[4].

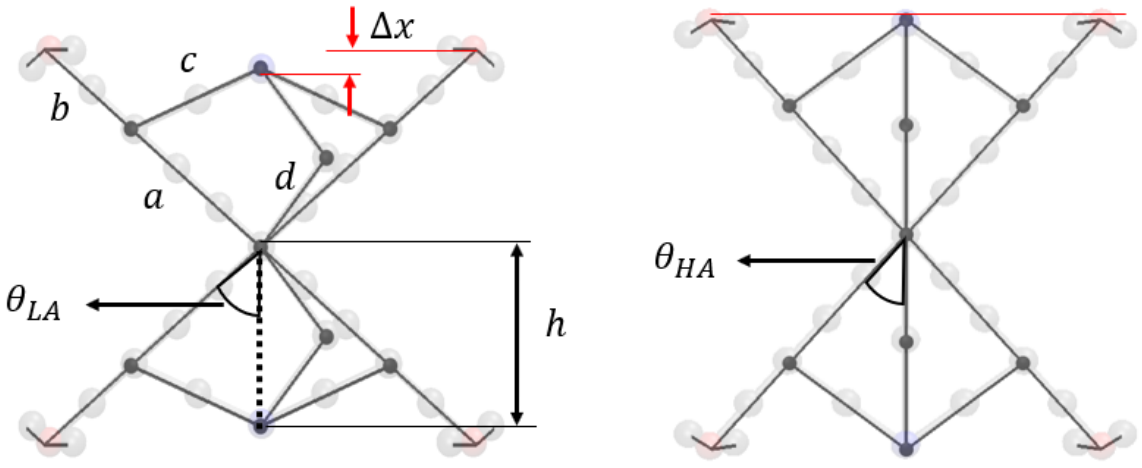

Supplementary Fig. 1 X-shaped nanoparticle geometry and its dimensions A) Open conformation B) Closed conformation

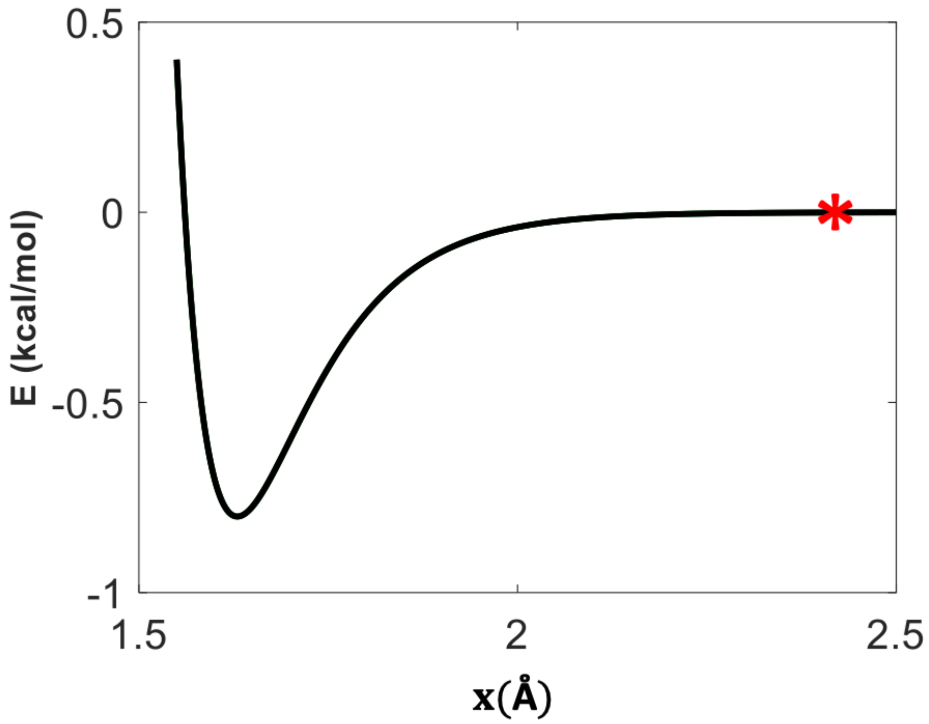

Supplementary Fig. 2 Energy landscape of the SBS interaction. The red star indicates the distance between SBS and the corresponding interaction energy when the dimer is in its LA state.

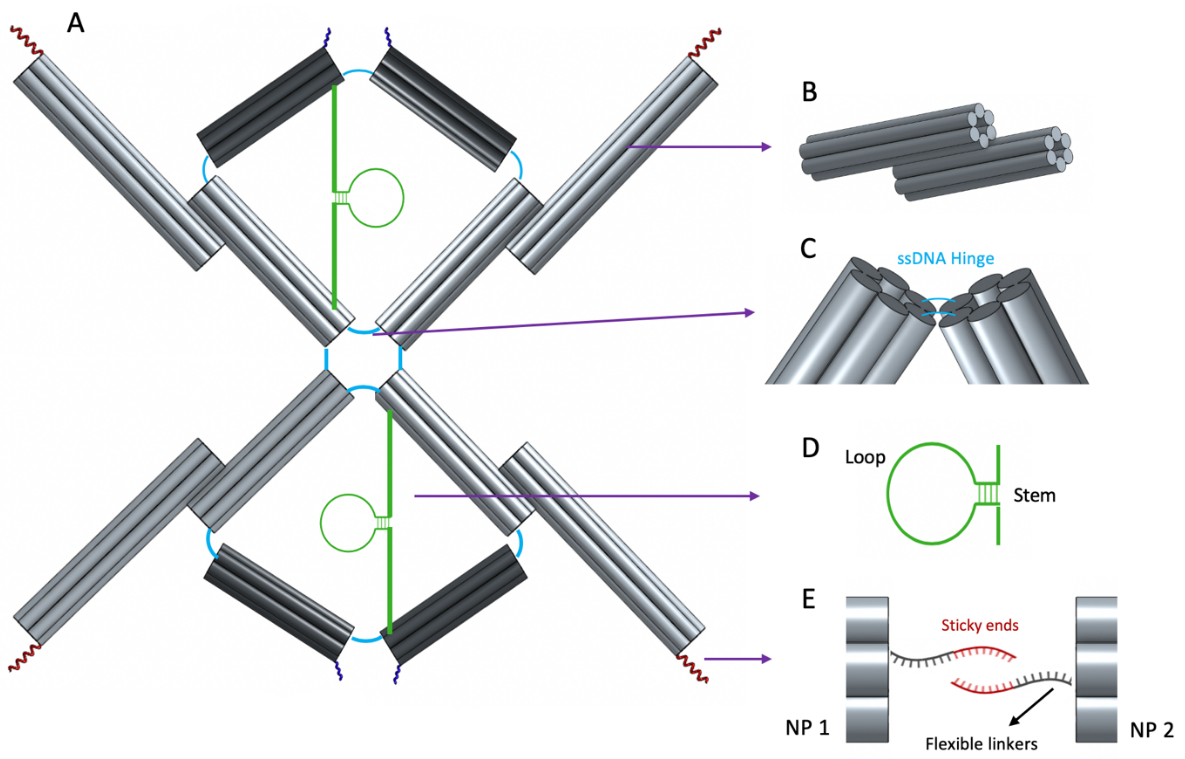

Supplementary Fig. 3 Possible DNA origami design of the X-shaped nanoparticle. A) Four main components of the nanoparticle are stiff members, hinges, switch and the interaction sites. These components can be created with B) double-stranded DNA helix bundles, C) single-strand DNA hinges, D) DNA hairpins, E) DNA overhangs with sticky ends. The members and helical appendages are color coded according to Figure 1.

Supplementary Table 1 Nanoparticle Dimensions

Member	Value
a	3.6 Å
b	2.4 Å
c	3.0 Å
d	2.24 Å

Supplementary References

1. Kauert, D. J., Kurth, T., Liedl, T. & Seidel, R. Direct Mechanical Measurements Reveal the Material Properties of Three-Dimensional DNA Origami. 5558–5563 (2011). doi:10.1021/nl203503s
2. Marras, A. E., Zhou, L., Su, H. & Castro, C. E. Programmable motion of DNA origami mechanisms. **112**, 713–718 (2015).
3. Ban, E. & Picu, C. R. Strength of DNA Sticky End Links. (2014). doi:10.1021/bm401425k
4. Maitra, A. & Arya, G. Model Accounting for the Effects of Pulling-Device Stiffness in the Analyses of Single-Molecule Force Measurements. **108301**, 1–4 (2010).
