# Supplementary material for: Self-strengthening biphasic nanoparticle assemblies with intrinsic catch bonds
Source: Nat Commun. 2021 Jan 4;12:85. doi: 10.1038/s41467-020-20344-4 (PMC7782701; doi:10.1038/s41467-020-20344-4)
Supplement: Supplementary file 3 — Description of Additional Supplementary Files [file 41467_2020_20344_MOESM3_ESM.pdf]

Description of Additional Supplementary Files

File Name: Supplementary Movie 1
Description: Trajectory of a sample MD simulation run of the dimer pulled with 140 pN, dimer separates with low affinity unbinding.

File Name: Supplementary Movie 2
Description: Trajectory of a sample MD simulation run of the dimer pulled with 180 pN, dimer separates with high affinity unbinding.

File Name: Supplementary Movie 3
Description: Replica exchange MD Simulation of self-assembly of nanoparticles
